# Treatment of Sindbis Virus-Infected Neurons with Antibody to E2 Alters Synthesis of Complete and nsP1-Expressing Defective Viral RNAs

**DOI:** 10.1128/mbio.02221-22

**Published:** 2022-09-07

**Authors:** Jane X. Yeh, Yunfan Fan, Maggie L. Bartlett, Xiaoyan Zhang, Norah Sadowski, Debra A. Hauer, Winston Timp, Diane E. Griffin

**Affiliations:** a Johns Hopkins Bloomberg School of Public Health, W. Harry Feinstone Department of Molecular Microbiology and Immunology, Baltimore, Maryland, USA; b Johns Hopkins University Whiting School of Engineering, Department of Biomedical Engineering, Baltimore, Maryland, USA; Columbia University Medical College

**Keywords:** alphavirus, defective viral genomes, nanopore sequencing, neuron infection, viral encephalitis, virus clearance

## Abstract

Alphaviruses are positive-sense RNA viruses that are important causes of viral encephalomyelitis. Sindbis virus (SINV), the prototype alphavirus, preferentially infects neurons in mice and is a model system for studying mechanisms of viral clearance from the nervous system. Antibody specific to the SINV E2 glycoprotein plays an important role in SINV clearance, and this effect is reproduced in cultures of infected mature neurons. To determine how anti-E2 antibody affects SINV RNA synthesis, Oxford Nanopore Technologies direct long-read RNA sequencing was used to sequence viral RNAs following antibody treatment of infected neurons. Differentiated AP-7 rat olfactory neuronal cells, an *in vitro* model for mature neurons, were infected with SINV and treated with anti-E2 antibody. Whole-cell RNA lysates were collected for sequencing of poly(A)-selected RNA 24, 48, and 72 h after infection. Three primary species of viral RNA were produced: genomic, subgenomic, and defective viral genomes (DVGs) encoding the RNA capping protein nsP1. Antibody treatment resulted in overall lower production of SINV RNA, decreased synthesis of subgenomic RNA relative to genomic RNA, and suppressed production of the nsP1 DVG. The nsP1 DVG was packaged into virus particles and could be translated. Because antibody-treated cells released a higher proportion of virions with noncapped genomes and transient transfection to express the nsP1 DVG improved viral RNA capping in antibody-treated cells, we postulate that one mechanism by which antibody inhibits SINV replication in neurons is to suppress DVG synthesis and thus decrease production of infectious virions containing capped genomes.

## INTRODUCTION

Alphaviruses (family *Togaviridae*) are important causes of viral encephalomyelitis worldwide and a significant public health concern due to their high mortality rate, potential for permanent neurologic damage, and limited availability of vaccines and therapeutics (1, 2). Alphaviruses are transmitted by mosquitoes and are expanding in worldwide geographic range due to climate change and globalization (3–6). Sindbis virus (SINV), the prototype alphavirus, has a positive-sense RNA genome of 11.7 kb that is 5′-capped, polyadenylated, and has two open reading frames: one encoding the nonstructural polyprotein that is subsequently processed into the individual nonstructural protein components (nsP1, nsP2, nsP3, and nsP4) and another encoding the structural polyprotein (capsid, E3, E1 and E2 envelope glycoproteins, 6K, and TF) (7).

**FIG 1:**
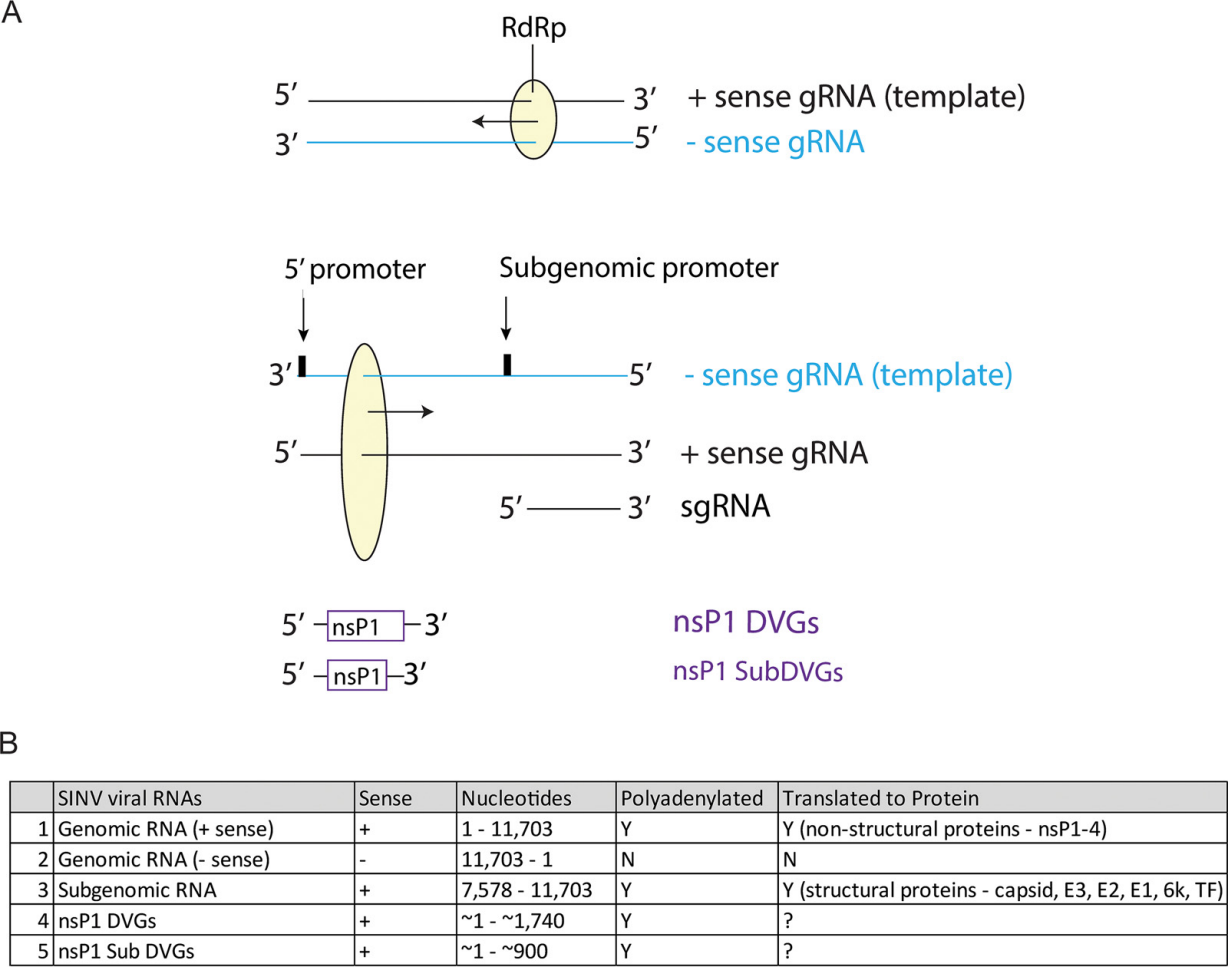
SINV RNA forms. (A) Schematic diagram of SINV RNA replication and the forms of SINV RNA made from each type of template strand. “gRNA” represents genomic RNA, “sgRNA” represents subgenomic RNA, and “DVG” represents the defective viral genome. (B) Table of the different SINV RNA forms indicating their sense, genome nucleotide composition, polyadenylation status, and translation to proteins.

In the SINV life cycle, 3 primary viral RNA species are made: (i) the full-length positive-sense genomic RNA (gRNA) that is eventually incorporated into released infectious virions, (ii) the negative-sense genomic RNA that serves as a template for viral RNA replication, and (iii) the shorter positive-sense subgenomic RNA (sgRNA), which includes the latter third of the genome encoding the structural proteins (Fig. 1). The relative production of these 3 viral RNAs changes over the course of the viral life cycle.

Upon infection, the positive-sense capped SINV RNA genome from the infecting virion is directly translated by host machinery to produce the nonstructural polyprotein P123 or P1234, depending on read-through of an opal stop codon between the nsP3 and nsP4 genes (10% occurrence) (8). nsP1 within the polyprotein anchors the replication complex to the plasma membrane and with nsP3 induces formation of the spherules in which RNA synthesis occurs (9). nsP4, the viral RNA-directed RNA polymerase (RdRp), is cleaved from P1234 and with P123 initiates synthesis of the negative-sense gRNA that serves as a template for production of more positive-sense viral RNAs. Initiation at the 5′ promoter of the negative-sense gRNA generates full-length positive-sense SINV gRNA, while initiation at the internal subgenomic promoter generates a shorter sgRNA that encodes the SINV structural proteins. Efficient transcription from the subgenomic promoter results in production of approximately 3-fold more sgRNA than gRNA, allowing for synthesis of more structural proteins that are incorporated into newly formed infectious virions (10). As the life cycle progresses, P123 is further cleaved by nsP2 into the individual nonstructural proteins nsP1, nsP2, and nsP3, and synthesis of negative-sense gRNA ceases while positive-sense RNA production continues (7).

In humans, SINV infection typically results in rash, fever, arthritis, and myalgia with persistent musculoskeletal pain (1). In rodents, SINV infection results in a well-characterized encephalomyelitis that parallels disease observed with alphavirus-induced encephalomyelitis in humans (2). Neurons of the brain and spinal cord are the primary targets of infection, with the olfactory tract, hippocampus, brainstem, and spinal cord motor neurons being especially susceptible (3, 4). SINV infection in rodents provides a model for investigation of alphaviral encephalomyelitis pathogenesis and recovery.

Following intranasal or intracranial infection with TE, a strain of SINV that does not cause fatal encephalomyelitis, weanling mice develop transient kyphoscoliosis, hind-limb paralysis, and muscular atrophy but recover in 2 to 3 weeks without lasting clinical or histological evidence of neuronal damage (5–8). Peak infectious viral titers in the brain and spinal cord are reached 3 to 5 days after infection, after which viral clearance begins (9). Infectious virus is no longer detectable in the central nervous system (CNS) by 7 to 8 days after infection; however, viral RNA levels decline much more slowly over weeks and continue to be detectable at low levels (10). This difference suggests that distinct mechanisms mediate clearance of infectious virus versus clearance of viral RNA from infected neurons, and both processes are essential to prevent continued or reactivated virus production.

Studies of SINV infection of immunodeficient mice have revealed many aspects of the immune responses involved in recovery. In particular, antibody against the SINV E2 glycoprotein is an important component of the immune response. Severe combined immunodeficient (SCID) mice that lack functional adaptive immune responses are unable to clear SINV from the CNS and continue to produce infectious virus (11). Adoptive transfer of monoclonal anti-E2 monoclonal antibody (MAb) alone is sufficient to induce clearance of infectious virus within 2 days after transfer (11). *In vitro* studies with primary rat dorsal root ganglion (DRG) neurons (11–13) and immortalized neuronal cell lines (14, 15) further revealed that antibody must be bivalent to be efficacious and does not require interaction with other cell types. Effects of antibody treatment of infected neuronal cells *in vitro* include decreased viral translation/transcription, inhibition of viral budding, restoration of host translation/transcription, restoration of host ability to respond to type I IFN, restoration of baseline intracellular Na^+^/K^+^ cation concentrations, and increased host cell survival (12, 14–16).

In differentiated rat AP-7 olfactory neuronal (dAP-7) cells (17, 18), antibody-mediated clearance of SINV is associated with initiation of a signaling cascade consisting of transient activation of NF-κB, induction of leukemia inhibitory factor (LIF) cytokine production, and subsequent prolonged activation of STAT3 that results in decreased synthesis of viral structural proteins and RNA and improved cell survival (16). Live-cell imaging of dAP-7 cells infected with recombinant SINV with Broccoli aptamer-tagged viral RNA also showed that anti-E2 antibody decreased overall viral RNA and improved cell survival (19). However, these studies were not able to determine the effects of antibody on the different viral RNAs.

Using high-throughput direct Oxford Nanopore MinION viral RNA sequencing, we evaluated the effect of anti-E2 antibody on RNA in SINV-infected dAP-7 olfactory sensory neuronal cells and found that antibody treatment induces changes in RNA abundance and type. Anti-E2 treatment improved production of rat RNAs and decreased production of SINV RNAs relative to untreated virus-infected cells. Antibody further affected the types of SINV RNAs produced, specifically decreasing sgRNA synthesis relative to gRNA synthesis and suppressing production of two previously unrecognized SINV RNAs containing the 5′ end, the nsP1 gene, and the 3′ untranscribed region (UTR) characteristic of deletion-type defective viral genomes (DVGs) (Fig. 1). Antibody treatment decreased the proportion of virions containing capped infectious gRNAs versus noncapped noninfectious gRNAs. The nsp1 DVGs could be translated and packaged, and we postulate these nsP1-containing DVGs have a proviral function through improved efficiency of SINV genome capping.

## RESULTS

### Nanopore sequencing of RNA from SINV-infected differentiated AP-7 neurons treated with antibody to E2.

To evaluate changes in SINV RNA at the nucleotide level following anti-E2 antibody treatment of infected dAP-7 cells, nanopore sequencing was employed. Nanopore sequencing directly sequences RNA to avoid potential biases from either PCR or reverse transcription and provides long reads for read-through of full-length viral genomic RNA (11.7 kb).

AP-7 cells were differentiated for 7 days into their nondividing form that models mature neurons (dAP-7 cells) (18, 20, 21) and then infected with the TE strain of SINV at a multiplicity of infection (MOI) of 10 based on plaques produced in BHK-21 cells, which results in initial infection of ~10% of the monolayer (18). Four hours after infection to allow for viral binding and entry, the cells were washed and treated with 5 μg/mL of monoclonal anti-E2 antibody, an amount within the range of antibody concentrations in brain (22), or medium alone as a negative control (untreated). At 24, 48, and 72 h after infection, total cellular RNA was collected and enriched for polyadenylated RNAs by oligo(dT)-conjugated magnetic bead sorting. The enriched mRNAs were then sequenced using a standard direct RNA nanopore method (Materials and Methods).

**FIG 2:**
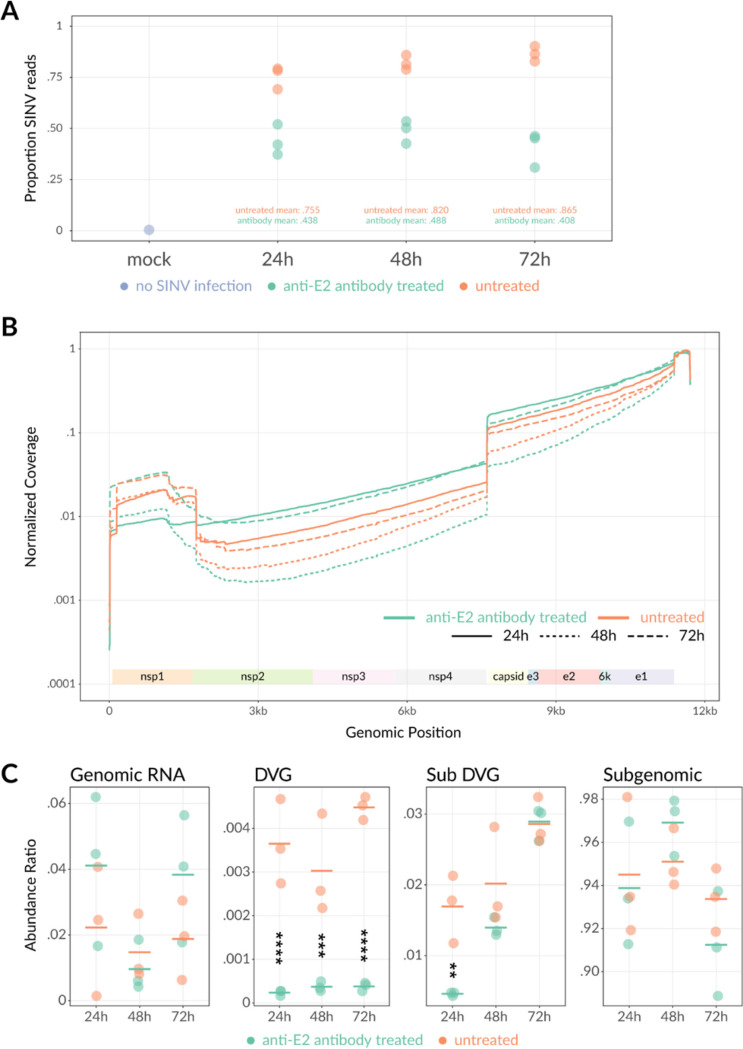
Nanopore sequencing of SINV-infected differentiated AP-7 cells with or without anti-E2 antibody treatment. Differentiated AP-7 cells were infected with SINV (MOI of 10) and mock treated or treated with anti-E2 antibody (5 μg/mL) 4 h after infection. At 24, 48, and 72 h after infection, total intracellular RNA was collected in triplicate, enriched for poly(A), directly sequenced via nanopore sequencing, and aligned to the rat or SINV genome. (A) Proportion of RNA reads that align to the rat versus SINV genome. Proportions indicate the number of rat or SINV reads divided by the total number of RNA reads at each time point for mock-infected cells. The mock-infected 24-h sample is indicated in purple. (All mock time points had 0 SINV-aligned reads.) SINV-infected no-antibody-treatment samples are indicated in orange, and SINV-infected antibody-treated samples are indicated in green. Each dot indicates a different biological replicate. (B) Averaged sequence coverage of SINV-aligned RNA reads. Coverage plots indicate relative sequencing depth normalized to run yield across the SINV genome with (green) or without (orange) antibody treatment at 24, 48, and 72 h after infection. Coverage data represent the average of 3 biological replicates. (C) Reads were classified into genomic, subgenomic, DVG, or subDVG SINV RNA species using coverage differences at junction locations (see Materials and Methods). Abundance ratios for each species were calculated as a proportion of total SINV reads for each sample. Each point represents 1 biological replicate, and horizontal lines indicate the mean from 3 biological replicates. **, *P* < 0.01; ***, *P* < 0.001; ****, *P* < 0.0001.

Sequencing reads were aligned to the rat and SINV genomes to assess host and viral gene expression. By 24 h after infection, 76% of the total RNA reads were aligned with the SINV genome in untreated virus-infected cells (Fig. 2A), reflecting the high efficiency with which SINV inhibits host transcription. By 48 and 72 h after infection, the percentages of SINV reads increased to >80% and >85% of the RNA sequenced, respectively. Similar to previous studies that suggested that anti-E2 antibody treatment reduces the effect of SINV-induced transcription inhibition (15), anti-E2 antibody treatment resulted in a greater proportion of rat reads and lower proportion of SINV reads at all three time points assessed (Fig. 2A). At 24 h, 44% of the RNA in E2 antibody-treated cells aligned to the SINV genome. At 48 h and 72 h, these proportions stabilized at 49% and 41%, respectively. Within the SINV-aligned reads, three primary SINV RNA forms were observed: (i) the 11.7-kb positive-sense full-length SINV gRNA (nucleotides [nt] 1 to 11703), (ii) the shorter 3.7-kb SINV sgRNA (nt 7646 to 11703), and (iii) a truncated RNA containing the nsP1 gene (nt ~70 to 1700), the 3′ UTR, and sometimes the 5′ UTR of the SINV genome (Fig. 2B). These truncated RNAs resemble deletion-type defective viral genomes (DVGs) in structure and are subsequently subcategorized as “DVGs” (with end between nt 1749 and 1755 of the SINV genome) or “subDVG” (with end at or before nt 1745) depending upon the viral RNA end. The negative-sense gRNA was not detected as it is not polyadenylated and would not be captured with the magnetic bead pulldown.

To evaluate changes in relative production of the different SINV RNA species with antibody treatment, we calculated the sequencing depth coverage across the SINV genome reference normalized to the total number of SINV reads in each run for each time point (Fig. 2B). We observed the expected 3′ bias due to the 3′→5′ directionality of nanopore sequencing as anchored to the poly(A) tail (23). Overall, coverage was greatest for the structural genes (capsid to E1) because this region is included in both the full-length SINV gRNA as well as the shorter sgRNA. Baseline levels of full-length SINV gRNA were reflected in the nonstructural gene region (nsP2 to nsP4) coverage. Coverage over both regions was relatively stable, consistent with synthesis of the SINV subgenomic and genomic RNAs as single molecules. The peaks in coverage at nsP1, the 3′ UTR, and the 5′ UTR reflect the separate truncated nsP1-containing DVGs identified.

To determine how the SINV RNAs were individually affected by antibody treatment, a viral RNA abundance ratio was calculated by taking the coverage differences at each junction site and dividing by the total of these differences (Fig. 2C). In general, gRNA levels were inversely correlated with sgRNA levels across the samples. Comparing the antibody-treated versus untreated cells, the relative abundance of gRNA was higher at 24 h and 72 h, but abundances were comparable at 48 h after infection; however, the differences were not statistically significant due to variation across the triplicate samples. By the same token, the sgRNA relative abundances were lower at the 24-h and 72-h time points with antibody treatment but higher at 48 h. These data suggest that anti-E2 antibody, in addition to decreasing overall viral RNA levels, also differentially affects the types of SINV RNA produced. Association of anti-E2 treatment with higher proportions of gRNA and lower sgRNA could be explained by a delay in the transition from gRNA to predominantly sgRNA production that occurs during late infection and would be a valuable topic of future investigation.

Antibody treatment also had a pronounced effect upon levels of the nsP1-containing DVGs. The antibody-treated cells had significantly lower DVG levels at all time points assessed (24 h, *P* < 0.0001; 48 h, *P* < 0.001; 72 h, *P* < 0.0001) and lower subDVG levels at 24 h (*P* < 0.01) than the untreated cells. These data suggest that in addition to antiviral antibody affecting the relative proportions of different SINV RNAs present, production of the nsP1-containing RNAs was especially sensitive to the effects of antibody.

### Evaluation of newly synthesized RNA from SINV-infected differentiated AP-7 neurons treated with antibody to E2.

**FIG 3:**
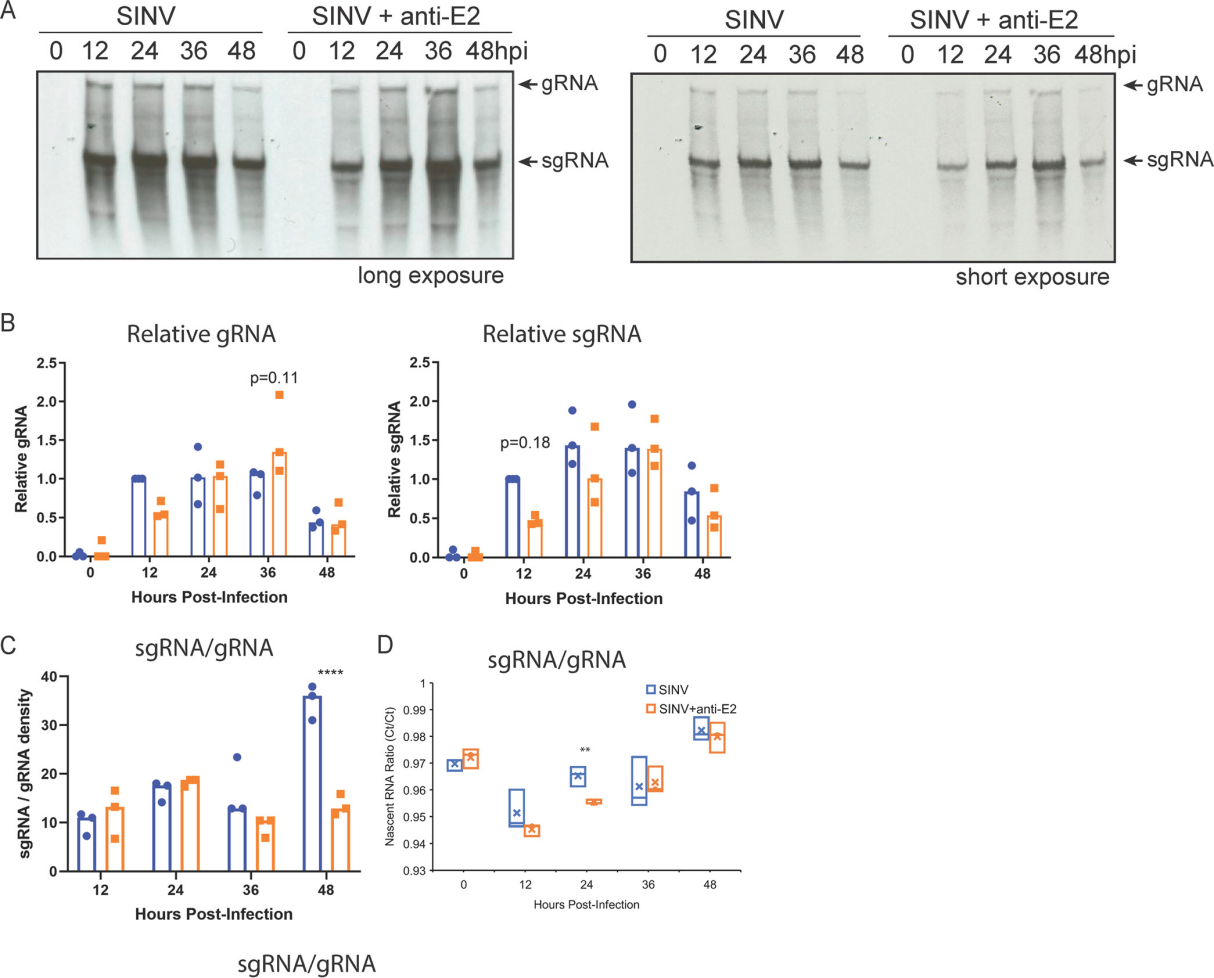
Anti-E2 antibody increases production of SINV genomic RNA and decreases production of subgenomic RNA. Analysis of newly synthesized viral RNA. Differentiated AP-7 cells were infected with SINV (MOI of 10) and treated with anti-E2 antibody (5 μg/mL) at 4 h after infection. At the indicated time points, cells were labeled with 20 μCi/mL [^3^H]uridine in the presence of dactinomycin (1 μg/mL) for 2 h. (A) Representative assessment of radioactive viral RNA synthesis by agarose-formaldehyde gel and autoradiography. (Top) Long exposure; (bottom) short exposure of the same gel. (B) Densities of labeled genomic RNA (gRNA [left]) and subgenomic RNA (sgRNA [right]) normalized to SINV-infected, untreated cells at 12 h; (C) ratio of relative subgenomic RNA to genomic RNA density; (D) ratios of qRT-PCR-quantified biotin-captured 5-ethynyl uridine-labeled nascent subgenomic RNA to genomic RNA in SINV-infected dAP-7 cells with and without antibody treatment. Data from three independent experiments are presented as mean ± SD. **, *P* < 0.01; ****, *P* < 0.0001.

The nanopore sequencing data showed that anti-E2 antibody treatment decreased levels of overall SINV RNA and affected the relative abundances of different SINV RNA species. To determine whether the antibody-induced changes reflect differences in RNA synthesis versus degradation and further investigate how shifts in viral RNA production are affected by antibody on a shorter time scale, dAP-7 cells were again infected with SINV (BHK-21 cell MOI of 10) and treated with medium (untreated) or 5 μg/mL anti-E2 antibody 4 h after infection (Fig. 3). At 0, 12, 24, 36, and 48 h after infection, the cells were pulsed with [^3^H]uridine for 2 h in the presence of dactinomycin to inhibit cellular transcription and allow for specific evaluation of viral RNA transcription. Whole-cell RNA was collected, separated by agarose-formaldehyde gel electrophoresis, and visualized by autoradiography.

Two distinct bands corresponding to the SINV gRNA and sgRNAs were detected (Fig. 3A) and quantified by densitometry (Fig. 3B). Twelve hours after infection, gRNA production over the 2-h pulse period was on average 2-fold higher in the SINV-infected untreated cells than in the antibody-treated cells, although the difference was not statistically significant (*P* = 0.25) (Fig. 3B). By 24 h, the amounts of gRNA were comparable. By 36 h after infection, gRNA production was 1.5-fold higher in the antibody-treated group than in the untreated group (*P* = 0.11). However, by 48 h, gRNA production declined in both the antibody-treated and untreated cells and the levels were roughly equivalent. sgRNA production over the 2-h pulse period was 2-fold lower at 12 h after infection in the antibody-treated versus untreated cells (*P* = 0.18), and at 24 h, production was 1.2-fold lower. By 36 and 48 h, levels of synthesis of sgRNA were comparable. To assess the relative proportions of viral RNAs being synthesized, the ratios of sgRNA to gRNA band density at each time point were compared between SINV-infected, untreated, and antibody-treated cells (Fig. 3C). While the ratios were comparable for the early time points, later time points showed that antibody treatment resulted in less sgRNA production. By 48 h after infection, the ratio of sgRNA to gRNA was 2.6-fold lower than in the untreated cells (*P* < 0.0001).

To better quantify changes in RNA synthesis induced by antibody treatment, cells were prelabeled with 5-ethynyl uridine for 15 h. RNA was extracted and nascent RNA isolated at the times indicated by reaction with biotin azide and streptavidin bead capture. Bead-captured viral RNAs were quantified by quantitative reverse transcription-PCR (qRT-PCR) using nsP2 primers (gRNA) and E2 primers (gRNA plus sgRNA), and ratios of nsP2 and E2 RNA expression were compared for treated and untreated infected cells (Fig. 3D). At early times, the ratios were similar, but at 24 h after infection, nascent sgRNA was more abundant in untreated cells than antibody-treated cells. These data recapitulate the trends observed of decreased sgRNA production with antibody treatment.

### SINV infection of dAP-7 cells results in production of a nsP1-containing defective genome.

**FIG 4:**
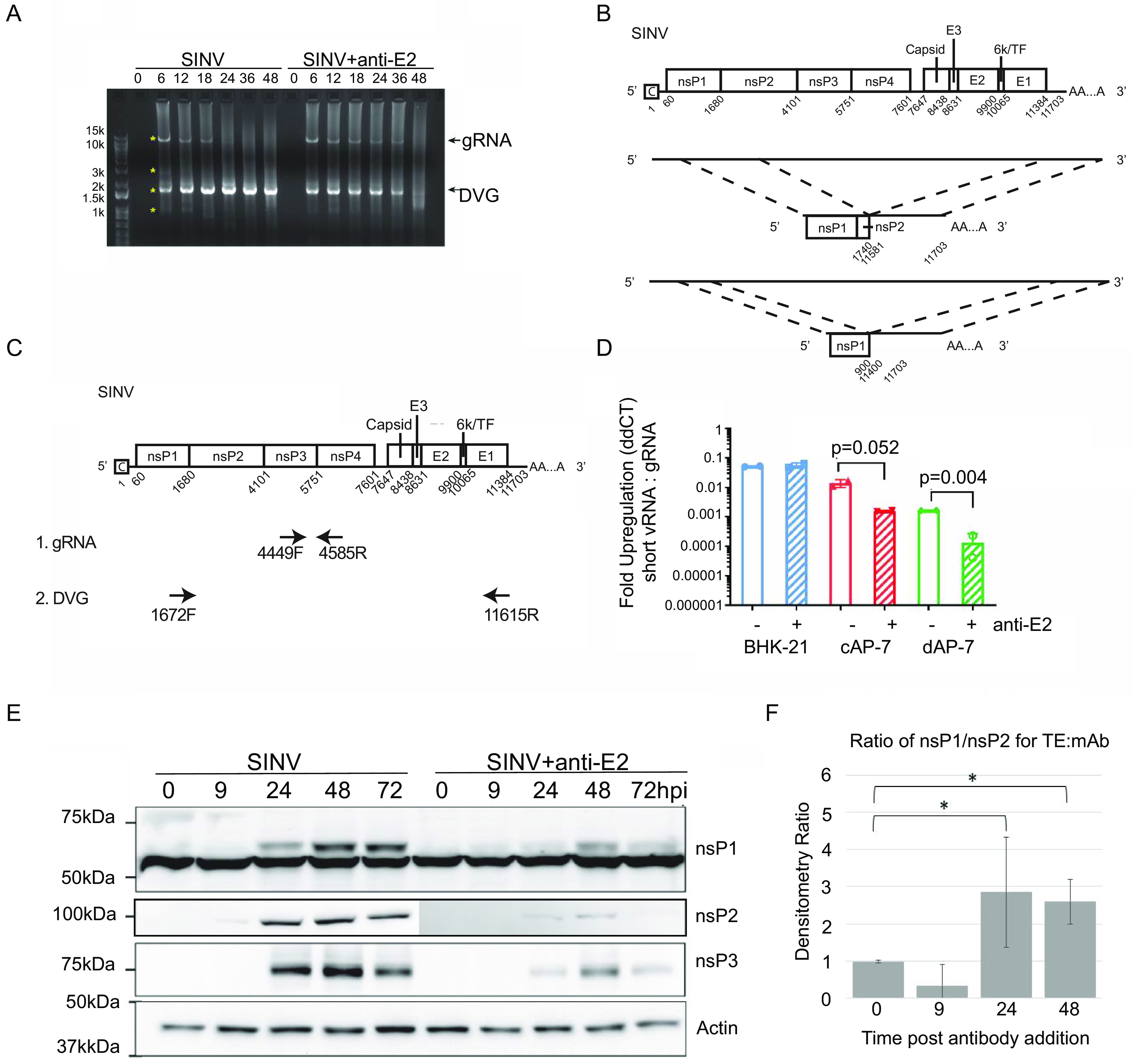
Antibody treatment increases SINV genomic RNA and decreases nsP1 defective viral genome RNA levels in dAP-7 cells. (A and B) Differentiated AP-7 cells were infected with SINV (MOI of 10) and treated with anti-E2 antibody (5 μg/mL) or medium (mock) at 4 h after infection. At the indicated time points, total cellular RNA lysates were collected. (A) Semiquantitative RT-PCR for SINV RNA using primers against the 5′ and 3′ ends of the SINV genome (SV-171F, SV-11655R). “gRNA” represents full-length SINV genomic RNA, and “DVG” represents the nsP1 defective viral genome. Asterisks indicate PCR bands excised for DNA extraction and Sanger sequencing. (B) SINV genome sequence alignment of the two defective viral genome sequences identified. (C and D) Differentiated AP-7 cells (dAP-7), undifferentiated cycling AP-7 cells (cAP-7), and BHK-21 cells were infected with SINV (MOI of 10) and treated with medium (mock) or anti-E2 antibody (5 μg/mL) at 4 h after infection. Twenty-four hours after infection, total cellular RNA was reverse transcribed to cDNA for qRT-PCR analysis of nsP1 defective viral genome levels. (C) Primer design used for qRT-PCR assay. Primers for the nsP1 defective viral genome span the deleted SINV region, while primers for the SINV genomic RNA are within the deleted region. (D) qRT-PCR analysis for nsP1 defective genome production. nsP1 RNA levels are expressed as fold regulation relative to SINV gRNA as calculated by ddCT. Data are presented as mean ± SD from two biological replicates. (E) Immunoblot of SINV nsP1, nsP2, and nsP3 expression from untreated and antibody-treated SINV-infected dAP-7 cells. (F) Densitometry was used to determine the ratios of nsP1 to nsP2 in immunoblots and to compare untreated (TE) to antibody-treated (MAb) SINV-infected dAP-7 cells. *, *P* < 0.05.

The nanopore sequencing data identified DVG-like SINV RNAs containing the nsP1 gene that were 1 to 2 kb in length and increased in abundance over time (Fig. 2B). To further characterize these RNAs, a PCR approach was used with primers against the SINV 5′ (SV171F) and 3′ (SV11655R) regions of the genome (Fig. 4). dAP-7 cells were infected with SINV (BHK-21 MOI of 10), and at 4 h after infection were treated with medium (mock) or 5 μg/mL anti-E2 antibody. At 0, 6, 12, 18, 24, 36, and 48 h after infection, total cellular RNA was collected and reverse transcribed to cDNA for PCR using random primers. The RT-PCR yielded two predominant bands: the first was ~11.7 kB (the size of full-length SINV gRNA), and the second was ~1.7 kB (the expected size of the nsP1 DVG) (Fig. 4A). The levels of the 11.7-kb SINV genome were higher and more sustained in the antibody-treated cells, while the levels of the 1.7-kb fragment were overall lower, consistent with trends of increased gRNA and decreased nsP1 DVG with antibody treatment observed in the sequencing (Fig. 2B) and RNA pulse-labeling (Fig. 3) experiments.

To confirm the identity of the PCR bands, 4 bands (the 2 predominant bands as well as 2 additional faint bands at 3 kb and 0.9 kb) from the untreated and antibody-treated 6- and 12-h lanes were excised and submitted for Sanger sequencing (Fig. 4A). Three of the bands (11.7, 1.7, and 0.9 kb) yielded sequencing results. The 11.7-kb band matched the full-length SINV genomic RNA. The remaining two bands were nsP1 gene-containing truncated SINV RNAs that matched DVG and subDVG sequences present in the nanopore sequencing data set. The most common and longer of the two DVGs included the nsP1 gene, a portion of the nsP2 gene, the 3′ UTR, and a poly(A) tail (Fig. 4B). The second nsP1 viral RNA was shorter and consisted of most of the nsP1 gene, the 3′ UTR, and a poly(A) tail. The presence of two nsP1 DVGs is consistent with the curved step-down shape of the nsP1 region peak in the sequencing coverage plots (Fig. 2B).

### Antiviral antibody treatment results in decreased nsP1 defective genomes in AP-7 neuronal cells but not BHK-21 cells.

To quantify levels of DVG and full-length SINV genomic RNA during infection, we designed a BRYT Green-based qPCR assay using primer sets that either span the deleted portion of the SINV genome for DVG amplification or are within the deleted portion (nsP3 gene) for SINV genomic RNA detection (Fig. 4C). Using this assay, we evaluated how antibody affects DVG and gRNA levels in BHK-21 cells, undifferentiated cycling AP-7 (cAP-7) cells that model immature neurons, and differentiated AP-7 (dAP-7 cells).

While anti-E2 antibody decreases virus production and improves viability in cell types that survive infection with SINV, such as differentiated neuronal cells (e.g., dAP-7 cells) or AT3-bcl-2 adenocarcinoma cells, it is not protective in cell types such as BHK-21, which are interferon-deficient, highly susceptible to SINV infection, and undergo rapid cell death following infection (11, 12, 15, 19). In cAP-7 cells, which express levels of innate immune genes such as IRF3 and IRF7 intermediate between BHK-21 and dAP-7 cells (20), SINV grows to intermediate titers, and antibody has some efficacy, but less than in dAP-7 cells. To determine whether the effect of anti-E2 antibody on nsP1 DVG production is host cell type specific and potentially related to its antiviral effects, BHK-21, cAP-7, and dAP-7 cells were infected with SINV (MOI of 10) and treated with anti-E2 antibody or media (untreated) at 4 h after infection. Twenty-four hours after infection, total cellular RNA was collected, transcribed to cDNA using random primers, and assessed for DVG and genomic RNA via qPCR. Levels of nsP1 DVG were evaluated as fold regulation of nsP1 DVG relative to SINV genomic RNA as determined by the threshold cycle (2^−ΔΔ^*^CT^*) method (24).

Infections of all three cell types generated detectable levels of nsP1 DVGs that correlated with the viral life cycle kinetics and levels of virus production. BHK-21 cells generated the highest relative amount of nsP1 DVG followed by cAP-7 and finally dAP-7 cells (Fig. 4D). Antibody treatment did not affect nsP1 DVG levels in the BHK-21 cells but significantly decreased nsP1 RNA production in both the cAP-7 and dAP-7 cells (Fig. 4D). These data confirm that nsP1 DVGs are products of the SINV life cycle and further suggest that the effects of anti-E2 antibody on nsP1 DVG production are cell type specific. It is possible, however, with faster growth of SINV in BHK-21 cells that earlier antibody treatment of infected BHK-21 cells may produce more comparable results.

Because nsPs are translated from gRNA as a polyprotein, they are present in equal amounts after processing. To determine whether the ratio of nsP1 to the other nsPs is affected by translation of the nsP1 DVG, lysates of infected dAP-7 cells with and without antibody treatment were probed for expression of nsP1, nsP2, and nsP3 (Fig. 4E). The production of all nsPs was suppressed by antibody treatment, but the ratio of nsP1 to nsP2 in untreated cells was greater than those in treated cells at 24 and 48 h after infection (Fig. 4F), suggesting that the nsP1 DVG may be translated.

### The nsP1 viral RNA-defective viral genome is packaged into particles and released.

DVGs are incomplete forms of the viral genome produced during infection due to errors in the viral genome replication process (25). The most common type of DVG involves deletion of an internal portion of the viral genome, as observed with the nsP1 SINV RNA (26, 27). DVGs can be propagated by complementation with wild-type virus and often replicate more rapidly than complete genomes due to their shorter length (28). In addition, if the DVG contains the viral genome packaging signals for association with capsid protein, it can be packaged into defective interfering particles (DIPs) and be transmitted to other cells for further propagation (28).

**FIG 5:**
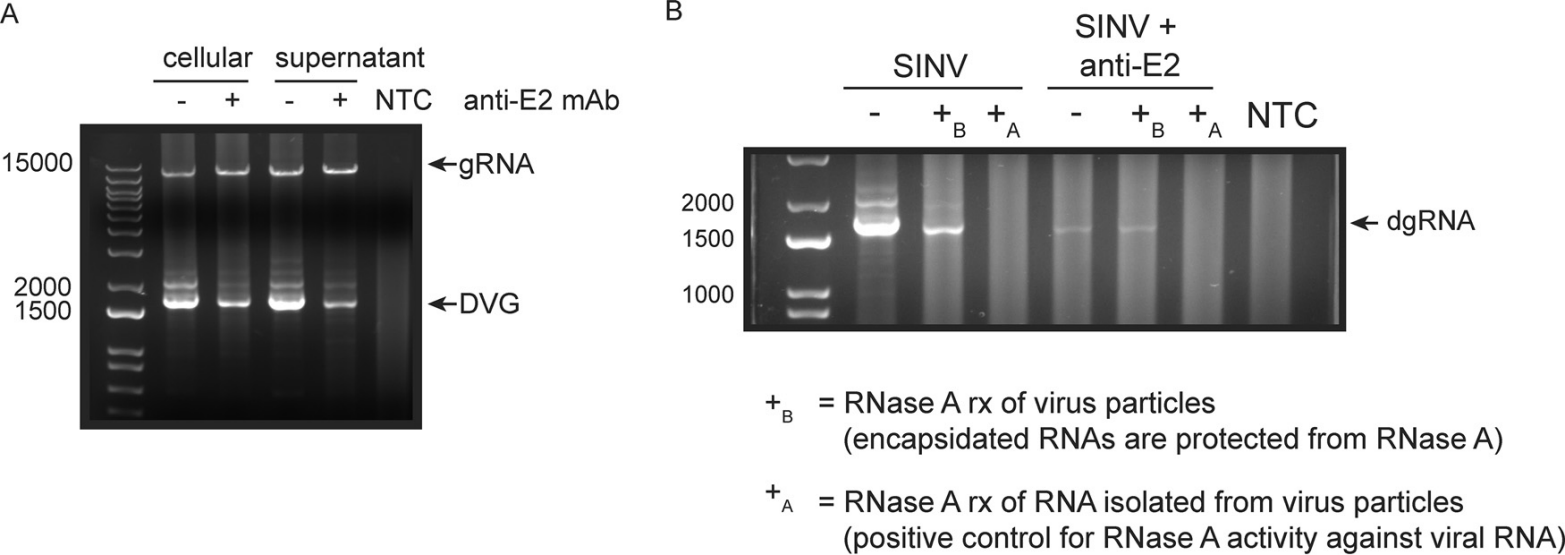
The nsP1 defective viral genome is packaged and released into viral particles. Differentiated AP-7 cells were infected with SINV (MOI of 10) and treated with medium (mock) or anti-E2 antibody (5 μg/mL) at 4 h after infection. Eighteen hours after infection, RNA from the cells and supernatant fluid was collected. (A) RNA was extracted from the cellular and supernatant samples. RT-PCR analysis for SINV genomic RNA (gRNA) and nsP1 defective viral genome RNA (DVG) was performed. “NTC” represents the no-template control. (B) RNase A protection assay. Culture supernatant was treated with RNase A (10 μg/mL) to digest all nonencapsidated RNAs (+_B_ [before RNA extraction]) or untreated (−). Following RNase treatment, RNA was extracted from the supernatant and reverse transcribed for RT-PCR analysis of nsP1 defective genome RNA (dgRNA). As a positive control for RNase activity, extracted RNA was also treated with RNase A under the same conditions (+_A_ [after RNA extraction]).

The SINV packaging signal required for viral RNA interaction with the capsid protein for assembly into viral particles includes nucleotides 945 and 1076 of the nsP1 gene (29), which are present within both nsP1 DVG sequences identified. To determine whether the nsP1 DVGs are packaged and possibly transmitted from cell to cell, we examined whether the nsP1 viral RNA is present in extracellular viral particles. dAP-7 cells were infected with SINV and treated with medium only (untreated) or medium containing anti-E2 antibody 4 h after infection, as previously described. Eighteen hours after infection, RNA was extracted from the cellular lysates and cell-free supernatant fluids for RT-PCR detection of the nsP1 DVGs using the same primers as before (SV171F and SV11615R) (Fig. 4A). The nsP1 DVG was detectable in both the cellular and supernatant fluid RNAs from the untreated and antibody-treated cells but was present at a lower level with antibody treatment (Fig. 5A). These data showed that the nsP1 viral RNA is released into the supernatant fluid during infection and confirmed the inhibitory effect of anti-E2 antibody on nsP1 DVG production.

To further confirm whether the nsP1 viral RNAs are encapsidated into viral particles, we treated culture supernatant fluids from SINV-infected and antibody-treated cells with RNase A to degrade unprotected single-stranded RNA leaving only encapsidated RNA. Following RNase treatment, RNA was extracted with TRIzol reagent that inhibits RNase activity for analysis. As a positive control for RNase activity, the TRIzol-extracted RNA was also directly treated with RNase A under the same conditions and then reextracted with TRIzol. The final isolated RNA was then assessed for nsP1 DVG using the RT-PCR approach from before.

For both the untreated and antibody-treated supernatant fluid samples, RNase A treatment did not completely remove all detectable nsP1 RNA, indicating that some of the nsP1 viral RNA was packaged (Fig. 5B). The positive control (RNase treatment after RNA isolation), however, showed complete nsP1 degradation, confirming that the nsP1 DVG is susceptible to RNase A degradation. Interestingly, RNase treatment did not affect levels of nsP1 RNA in the antibody-treated cells, suggesting that all released nsP1 RNA is encapsidated. This could occur by antibody prolonging survival of infected dAP-7 cells, thereby delaying deterioration of the host cell and release of intracellular viral RNA.

### The nsP1 defective viral genome can be translated.

Because the nsP1 DVG sequence can include the SINV 5′ terminus, 3′ UTR, and a poly(A) tail, components necessary for recognition by the host translation machinery, we next asked whether the DVGs could be translated into a viral protein. To answer this question, nsP1 DVG cDNA sequences were cloned into the pcDNA3.1/V5-His-TOPO expression vector with an upstream SP6 promoter for *in vitro* transcription and protein expression. Only the shorter of the two nsP1 DVGs (subDVG) without a poly(A) tail was successfully cloned due to Escherichia coli toxicity. This DNA sequence was *in vitro* transcribed to RNA, and a poly(A) tail was subsequently added using poly(A) polymerase. The resulting RNA was transfected into uninfected undifferentiated cAP-7 cells that are more efficiently transfected than dAP-7 cells. RNA transcribed from the pTE plasmid that contains the complete cDNA sequence of SINV TE was transfected in parallel as a positive control, and the nsP1 RNA sequence without a poly(A) tail was included as a negative control. Twenty-four hours after transfection, protein lysates were collected and evaluated by immunoblotting using polyclonal antibody against SINV nsP1.

**FIG 6:**
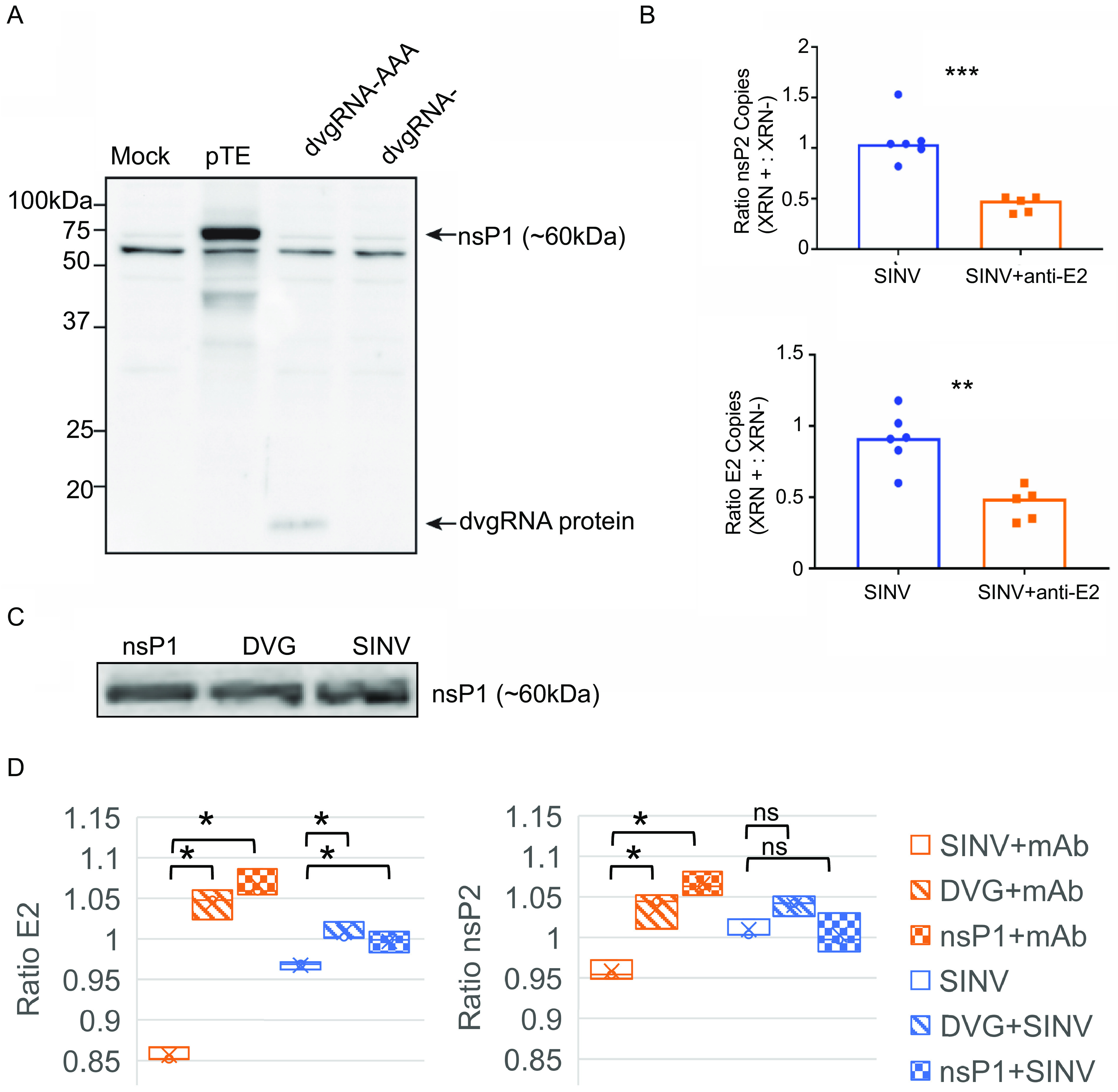
The nsP1 defective viral genome RNA can be translated, and expression is associated with increased SINV RNA capping efficiency. (A) The shorter nsP1 defective viral genome RNA (subDVG) was cloned into the pcDNA3.1 DNA expression vector and *in vitro* transcribed to RNA (dvgRNA). The RNA was capped and polyadenylated *in vitro*, purified, and transfected into uninfected undifferentiated AP-7 cells (dvgRNA-AAA). As controls, pTE (complete SINV plasmid) and unpolyadenylated defective viral genome RNA (dvgRNA−) were also transfected. Twenty-four hours after transfection, lysates were collected and assessed by immunoblot probing with a polyclonal antibody against SINV nsP1. (B) Proportion of capped genomes in virus particles released from untreated and antibody-treated SINV-infected dAP-7 cells. Differentiated AP-7 cells were infected with SINV (MOI of 10) and treated with medium (mock) or anti-E2 antibody (5 μg/mL) 4 h after infection. Twenty-four hours after infection, virus particles were purified from the supernatant fluid by ultracentrifugation and viral RNA was extracted and treated with XRN-1 exoribonuclease to degrade noncapped RNAs (XRN +) or left untreated (XRN −). Following treatment, the RNA was reverse transcribed to cDNA and assessed by qRT-PCR for SINV nsP2 and E2 transcripts. The *y* axis indicates the ratio of copy numbers of nsP2 or E2 transcripts in the XRN-treated versus untreated groups. (C) Immunoblot of nsP1 protein expression 24 h after SINV infection or transfection with pGenLenti vectors expressing nsP1 or nsP1 DVG; (D) proportion of capped genomes in virus particles released from untreated (blue) or antibody-treated (red) cells transiently transfected with lentivirus vector plasmids expressing either nsP1 or nsP1 DVG 24 h prior to infection with SINV. Virions were isolated with Viraffinity reagent, and RNA capping was analyzed using XRN degradation, as described above. Data are presented as mean ± SD and are from three biological replicates. *, *P* < 0.05; **, *P* < 0.01; ***, *P* < 0.001.

pTE *in vitro*-transcribed RNA induced robust expression of SINV nsP1 protein at the expected molecular weight of ~60 kDa (Fig. 6A). The lighter band at the slightly lower molecular weight of ~55 kDa was due to antibody nonspecific reactivity and was also present in mock-transfected cells. The nsP1 DVG RNA without a poly(A) tail (dvgRNA^−^) did not induce protein expression and was identical to the mock-transfected cells. The polyadenylated nsP1 RNA-transfected cells uniquely produced a protein band detectable at a significantly lower molecular weight (15 kDa) than expected (60 kDa), suggesting that the shorter nsP1 DVG results in production of a truncated form of nsP1. Why the protein differs so much in size from the full-length nsP1 protein despite the DVG containing most of the nsP1 sequence is unclear and may be due to the absence of other components from the SINV genome.

### Anti-E2 antibody treatment decreases efficiency of SINV genome capping.

We next explored what biological function the nsP1 DVG protein product might have in SINV infection. Due to the correlation between decreased viral replication and decreased nsP1 DVG production, we hypothesized that the nsP1 DVG may be playing a proviral role that is inhibited by anti-E2 antibody.

The SINV nsP1 protein has methyltransferase and guanylyltransferase activity, and in cooperation with nsP2, produces the RNA 5′ type 0 ^7me^GpppA cap required for translation (30, 31). However, not all SINV RNAs are capped. Early in infection of BHK-21 cells, released viral particles contain a high proportion of noncapped SINV gRNAs that are noninfectious. Later in infection, the proportion of released virions containing capped SINV gRNAs increases, resulting in mostly infectious virions (32, 33). We hypothesized that nsP1 DVG production and cell-to-cell transmission via DVG-containing particles could be involved in this change by allowing for greater synthesis of nsP1 protein for RNA capping during late infection. Antiviral antibody treatment, by suppressing nsP1 DVG production and neutralizing nsP1 DVG-containing particles, would lead to less nsP1 protein, decreased SINV RNA capping efficiency, and therefore decreased infectious virus production.

To assess whether antibody treatment affects the proportion of viral particles that have capped genomes, we performed an exoribonuclease degradation assay utilizing the enzyme XRN-1, which selectively degrades noncapped RNAs. Virus particles from the supernatant fluids of SINV-infected, untreated, and antibody-treated dAP-7 cells 24 h after infection were purified by ultracentrifugation. The RNA was isolated from the virus particles using TRIzol reagent and treated with XRN-1 for 1 h at 37°C to degrade noncapped RNAs. The remaining XRN-resistant RNAs were extracted using phenol-chloroform and reverse transcribed to cDNA for SINV RNA qRT-PCR analysis. The ratio of SINV nsP2 and E2 RNA with or without XRN-1 treatment was compared. Levels of nsP2 and E2 RNA from the SINV-infected, untreated cells were not affected by XRN-1 treatment (ratio of ~1) (Fig. 6B), suggesting that the majority of the packaged SINV genomic RNA is capped at 24 h after infection in dAP-7 cells. In contrast, with anti-E2 antibody treatment, roughly 50% of the virion RNA was susceptible to XRN-1 degradation (64% decrease in nsP2 relative to untreated cells, *P* < 0.001; 46% decrease in E2, *P* < 0.001), suggesting that approximately 50% of the packaged SINV RNA was not capped.

To confirm the effects of supplemental nsP1 on genome capping and assess the effects of additional nsP1 expression in antibody-treated cells, dAP-7 cells were transiently transfected with lentiviral vector plasmids expressing nsP1 or the nsP1 DVG (Fig. 6C), infected with SINV and then treated or not with anti-E2 antibody. In cells transfected with either DVG or nsP1, the antibody-mediated reduction in capped genomes was reversed to resemble that of untreated cells, further supporting a role for the nsP1 DVG in increasing SINV RNA capping efficiency (Fig. 6D).

## DISCUSSION

**FIG 7:**
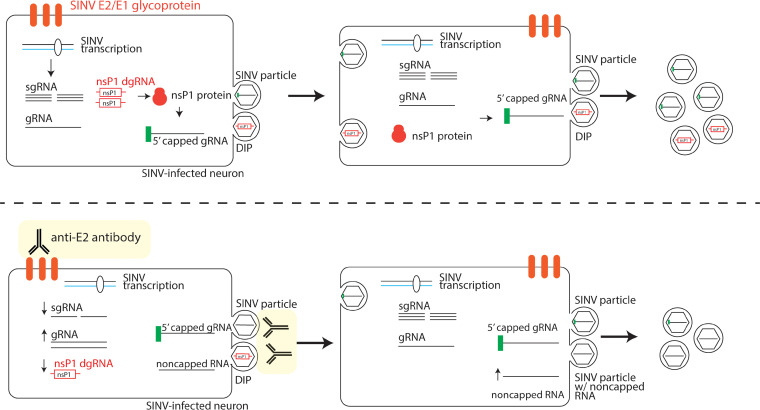
Model of nsP1 defective genome proviral activity (above) and interactions with antiviral anti-E2 antibody (below). nsP1 DVG expression results in increased production of SINV nsP1 protein and improved SINV RNA capping efficiency. The nsP1 DVG is packaged into defective interfering particles (DIPs) that can spread from cell to cell and replicate during coinfection with wild-type virus. Anti-E2 antibody suppresses viral RNA transcription and particularly affects SINV sgRNA and nsP1 DVG production. Anti-E2 antibody also inhibits viral and DIP budding by direct binding of surface E2. Inhibition of spread of the nsP1 DVG by inhibition of budding or neutralization contributes to overall decreased viral replication by decreasing production of nsP1 protein and decreasing SINV RNA capping efficiency during late infection.

*In vitro* treatment of SINV-infected neuronal cells with bivalent antibody to the E2 glycoprotein inhibits virus replication, restores cellular functions, blocks virus release, and improves neuron viability (11, 12, 14–16, 19). In this study, we used Oxford Nanopore MinION direct RNA sequencing to analyze changes at the RNA level in SINV-infected differentiated AP-7 rat sensory olfactory neurons that were or were not treated with anti-E2 antibody. Antibody treatment decreased overall levels of viral RNA and altered the proportions of different SINV RNA forms. Genomic RNA levels were relatively higher with antibody treatment, while sgRNA levels were lower, consistent with the previously reported antibody-mediated inhibition of structural protein synthesis (16). In addition, antibody suppressed production of two previously unrecognized DVGs encoding the nsP1 capping protein. The nsP1 DVGs could be translated and packaged into extracellular particles. Assessment of the proportion of noncapped RNAs in extracellular virus particles by XRN-1 degradation revealed that 24 h after infection the majority of RNAs from released SINV particles were capped. With antibody treatment, however, only 50% of the virus particle RNA was capped but exogenous expression of nsP1 improved capping efficiency. These data suggest that the DVGs increase the amounts of nsP1 protein produced to improve viral RNA capping and virion infectivity. In addition, we postulate that anti-E2 antibody inhibits SINV replication by decreasing production of nsP1 DVG-containing particles for cell-to-cell transmission and decreasing production of infectious SINV particles containing capped genomic RNA (Fig. 7).

The protective effects of anti-E2 antibody are multifactorial and induced by physical binding of antibody to the surface of infected cells and the intracellular signaling cascade triggered (16). During infection SINV induces loss of membrane potential following inhibition of Na^+^ K^+^ ATPase activity (15, 34), shutoff of host protein synthesis due in part to activation of RNA-dependent protein kinase (PKR) (18), and apoptotic cell death (35). Although complete mechanisms are not yet established, antiviral antibody partially reverses or inhibits these effects by restoring intracellular cation concentrations and host protein synthesis. Intracellular Na^+^ and K^+^ concentrations affect interaction of the viral nsP4 RNA polymerase with the genomic and subgenomic promoters; therefore, restoration of intracellular cation concentrations results in restored host protein synthesis and improved interferon and innate response factor production (14). In addition, antibody binding to viral E2 protein on the surface of infected cells induces activation of the NF-κB signaling and STAT3 cell survival pathways that initiate cellular responses that inhibit amplification of early virus replication complexes (15, 16).

In this study, we observed that not all viral RNAs were similarly affected by antibody treatment, and synthesis of gRNA was transiently improved. This is consistent with previously observed delayed processing of the nonstructural polyprotein with antibody treatment that may prolong transcription of the negative-sense genome template and delay the shift to exclusive production of the positive-sense SINV RNAs (15). Translation of viral gRNA requires the same machinery and regulation as translation of cellular mRNAs (36). Because synthesis of the negative-strand genomic RNA template requires continued production of the nonstructural polyprotein (37), maintaining host protein synthesis would also prolong the synthesis of the negative-strand template leading to greater genomic RNA replication. Although of interest, levels of negative-sense gRNA were not specifically evaluated in the nanopore sequencing analysis because it is not polyadenylated or in the pulse-labeling experiments because it is not distinguishable in size from positive-sense gRNA. Future studies that utilize next-generation techniques such as SLAM-seq (thiol-linked alkylation for metabolic sequencing of RNA) to more accurately quantify RNA synthesis during SINV infection with or without antibody treatment will be necessary to confirm these findings. Furthermore, the mechanism for antibody-mediated modulation of viral RNA synthesis is unknown. It is possible that antibody affects the availability of cellular factors that regulate transcription within neurons required for viral RNA synthesis and will require a more comprehensive analysis of the transcriptional and proteomic changes induced by antibody treatment of infected cells.

In contrast to gRNA, antibody decreased production of SINV sgRNA early in infection, consistent with the previously described antibody-induced decrease in structural protein synthesis (16). One possible explanation is that restoration of host protein synthesis with antibody treatment may allow production of host antiviral factors that decrease the efficiency of viral transcription, especially from the subgenomic promoter. High-throughput interactome studies have shown that hundreds of mammalian proteins are capable of binding SINV RNA to regulate both transcription and translation (38, 39). Therefore, studies of antibody-induced changes in expression of these RNA-binding proteins, especially those that bind the subgenomic promoter region, would also be a valuable area of investigation.

These studies revealed that SINV infection of BHK-21 cells, as well as dAP-7 neuronal cells, resulted in production of previously unrecognized DVGs consisting of the SINV nsP1 gene, sometimes a portion of the nsP2 gene, the 3′ UTR, and a poly(A) tail. Similar deletion-type DVGs have been reported for many families of RNA viruses, including alphaviruses (26, 27, 40, 41). DVGs are products of errors in the viral transcription process and generally inhibitory to wild-type virus replication due to competition for viral/host resources and activation of the host immune response. However, for some viruses, DVGs facilitate infection or persistence (40). In Sendai virus and respiratory syncytial virus infections, high intracellular levels of DVGs protect infected cells from death by altering the outcome of the mitochondrial antiviral signaling-mediated tumor necrosis factor response (42). With hepatitis C virus infection, coexpression of a wild-type virus and DVG results in increased viral replication, suggesting an important biological role for the DVG (43). In our study, the specific accumulation of the nsP1 DVGs suggests that they selectively replicate and may enhance SINV replication in neurons. Observations that further support the possibility that the nsP1 DVGs may play a proviral role in SINV infection include (i) packaging into enveloped particles with the possibility of cell-to-cell spread, (ii) DVG translation into protein, and (iii) association with increased SINV RNA capping.

nsP1 has an amphipathic peptide segment and is palmitoylated for binding to the plasma membrane where it induces formation of spherules that organize replication complexes for viral RNA synthesis. nsP1 oligomerizes to form a dodecameric ring at the neck of the spherules to cap RNAs as they are exported (44–46). Genomic RNA needs to be capped for translation of the nonstructural polyprotein, formation of replication complexes, and protection from degradation, so virions containing noncapped genomes are not infectious (33). However, there may be other functions for noncapped viral RNA as analysis of nsP1 mutants with altered capping efficiency has shown that increased capping decreases virus replication in BHK-21 cells by impairing virion production (47). Antiviral antibody treatment suppressed production of the nsP1 DVG and was associated with a higher proportion of noncapped SINV genomes packaged into viral particles than untreated SINV-infected cells. Transfection of infected antibody-treated dAP-7 cells with vectors expressing nsP1 or the nsP1 DVG restored the proportion of capped to noncapped SINV genomes to those present in untreated SINV-infected cells. However, cell type likely substantially contributes to these observations and the relative roles of capped and noncapped RNAs. SINV replication in mature neurons (e.g., dAP-7 cells) is highly restricted compared to replication in BHK-21 cells or immature neurons and virus replication in BHK-21 cells is not decreased by treatment with anti-E2 antibody, so optimal production of infectious particles may be more important in dAP-7 cells than BHK-21 cells (18, 19). In addition, we hypothesize that antibody may neutralize infectivity of defective particles containing the nsP1 DVG, thereby preventing their entry into and propagation in new cells.

In addition to its role in RNA synthesis, nsP1 can induce filopodia to facilitate cell-to-cell virus transmission, a property of potential importance for transsynaptic neuronal spread (48–51). nsP1 can interfere with interferon induction and signaling to influence virulence (52–56), and increased capping efficiency is associated with decreased production of inflammatory cytokines, with consequences for neurovirulence (57–59). *In vivo*, antibody clears infectious virus from SINV-infected brain and spinal cord neurons of mice within 8 days after infection, but viral RNA is eliminated much more slowly and continues to be detectable for months (9–11, 60). Future studies to determine whether the nsP1 DVG is produced *in vivo* and, if so, whether levels are regulated by antibody within the CNS will be of interest.

## MATERIALS AND METHODS

### Cell culture.

AP-7 rat olfactory sensory neuronal cells immortalized with a temperature-sensitive simian virus 40 (SV40) T antigen (gift from Dale Hunter, Tufts University, Boston, MA) (21) were grown under the permissive conditions of 33°C and 7% CO_2_ in Dulbecco’s modified Eagle’s medium (DMEM) (Gibco) supplemented with 10% heat-inactivated fetal bovine serum (FBS) (Atlanta Biologicals), 100 U/mL penicillin, 100 μg/mL streptomycin, and 2 mM glutamine (Gibco). At 25% confluence, cells were differentiated for 7 days by shifting the cultures to 39°C and 5% CO_2_ and supplementing the medium with 1 μg/mL insulin, 20 μM dopamine, and 100 μM ascorbic acid (Sigma). Cells were routinely tested for mycoplasma using the MycoAlert PLUS mycoplasma detection kit (Lonza).

### Virus infection and antibody treatment.

Stocks of the TE strain of SINV (5) were generated from RNA transcribed from cDNA, transfected into and assayed by plaque formation on BHK-21 cells. Differentiated AP-7 cells were infected at a BHK-21 cell multiplicity of infection (MOI) of 10 in DMEM–1% FBS for 1 h. IgG from the supernatant fluids of the SV127 antibody hybridoma clone (mouse monoclonal IgG3 against the SINV E2 glycoprotein) (12, 61) was purified by adsorption to protein G and resuspended in phosphate-buffered saline (PBS) (Genscript Hybridoma Cell Culture Antibody Production Services). Four hours after infection, cell cultures were washed to remove unbound virus and treated with 5 μg/mL of antibody diluted in DMEM–1%FBS. Following 1 h of incubation, the antibody was diluted to 1.25 μg/mL by the addition of 3 volumes of DMEM–1%FBS and maintained until sample collection.

### RNA isolation, RT-PCR, and qRT-PCR for SINV RNA.

Cellular RNA samples were collected by direct lysis in TRIzol reagent (Thermo Fisher Scientific), and supernatant fluid RNA samples were collected by adding 1 mL TRIzol to 100 μL culture fluid. RNA was purified according to manufacturer’s instructions and reverse transcribed into cDNA using (i) the Maxima H minus first strand cDNA synthesis kit (Thermo Fisher Scientific) with oligo(dT) primers for RT-PCR or (ii) a high-capacity cDNA reverse transcription kit (Applied Biosystems) with random primers for qRT-PCR. For PCR amplification of SINV RNA for gel electrophoresis, Phusion polymerase (Thermo Fisher Scientific) and the following primers were used for PCR: 171F (5′-GACCATGCTAATGCCAGAGC-3′) and 11655R (5′-GTTATGCAGACGCTGCGTGG-3′). For sequencing, the band of interest was extracted using the QIAquick gel extraction kit (Qiagen) and submitted to the Johns Hopkins Synthesis and Sequencing Facility. To quantify levels of SINV gRNA versus nsP1 DVG, qRT-PCR was performed on the cDNA samples using the GoTaq master mix (Promega) and the following primers on a 7500 Fast real-time PCR system: for detection of the nsP1 defective genome, 1672F (5′-TCGGAGCAGCATTAGTTGAA-3′) and 11615R (5′-ATTATGCACCACGCTTCCTC-3′); for detection of the SINV genomic RNA, 4449F (5′-GGAAAAGACCGCCTTGAAGT-3′) and 4585R (5′-ACAGACTCCTTAAGTTGGAGTGC-3′). To quantify levels of the SINV nsP2 and E2 genes, qRT-PCR was performed using EagleTaq universal master mix (Roche) and the following sequences: for SINV nsP2, nsP2 3373F, 5′-CCG CAA GTA TGG GTA CGA TCA-3′, and nsP2 3454R, 5′-GTG CCC TTC CCA GCT AGC T-3′; for TaqMan probe nsP2 3317, 5′-FAM (6-carboxyfluorescein)-CCA TTG CCG CCG AAC TCT CCC-TAMRA (6-carboxytetramethylrhodamine)-3′; for SINV E2, E2 8732F, 5′-TGG GAC GAA GCG GAC GAT AA-3′, and E2-8805R, 5′-CTG CTC CGC TTT GGT CGT AT-3′; and for TaqMan probe E2 8760, 5′-FAM-CGC ATA CAG ACT TCC GCC CAG T-TAMRA-3′.

### Nanopore sequencing of RNA.

For direct sequencing of the RNA, poly(A) RNAs were isolated from frozen cell pellets using TRI-Reagent (Invitrogen), following the manufacturer’s instructions, with 1-bromo-3-chloro-propane for phase separation. Five to 50 μg of total cellular RNA diluted in 50 μL of nuclease-free water was poly(A) enriched using the NEXTflex poly(A) beads (BIOO Scientific). The resulting poly(A) RNAs were eluted in nuclease-free water and quantified using the Qubit 4 fluorometer (Thermo Fisher Scientific). One hundred forty to 800 ng of RNA was prepared for nanopore direct RNA sequencing following the ONT SQK-RNA002 kit protocol, including the optional ONT recommended reverse transcription step using Superscript IV (ThermoFisher). RNA sequencing was performed on the Oxford MinION platform using ONT R9.4.1 flow cells. The ONT Guppy workflow (version 4.0.11) was used for RNA base calling. Strand reads that had an average sequence quality of 7 or higher were classified as passing quality reads. Minimap 2 version 2.17 was used to align the nanopore RNA reads to the rat genome Rnor_6.0 and SINV TE strain genome (NC_001547.1). The junction sites of subgenomic RNA and the DVGs were determined to be positions 7609 and 1751, respectively, and the abundance of each species was defined as the coverage difference at these junctions. The abundance of genomic RNAs for each sample was defined as the coverage at position 1752. Because the subDVG does not have a sharp 3′ junction, the abundance of subDVG RNA is calculated as the difference between the coverage at position 1110 and the minimum coverage between 1111 and the DVG junction.

### RNA labeling.

For radioactive isotope labeling of newly synthesized RNA, dAP-7 cells were incubated at the indicated times after infection with DMEM containing [5,6-^3^H]uridine (20 μCi/mL) (Perkin Elmer) and dactinomycin (1 μg/mL) (Sigma) at 39°C for 2 h. Cellular lysates were collected with TRIzol and RNA purified as described above. RNA concentrations were determined by NanoDrop spectrophotometer. Two-microgram RNA samples were prepared for electrophoresis with RNA gel loading dye (Thermo Fisher Scientific) and separated on a 1% formaldehyde-agarose gel. Fluorography was performed with 2.5% 2,5-diphenyloxazole as previously described, and the gels were dried and autoradiographed at −80°C (62). Densitometric analysis was performed using ImageJ software.

For alkyne-reactive uridine labeling and subsequent capture of newly synthesized RNA, the Click-iT nascent RNA capture kit (Thermo Fisher) was used. In brief, dAP-7 cells were cultured with DMEM containing 5-ethynyl uridine (0.2 mM) for 15 h, and at indicated times after infection with or without antibody treatment, RNA was isolated and biotin azide was clicked onto nascent RNA for capture with streptavidin. One microgram of RNA was heated at 68 to 70°C for 5 min and incubated with Dynabeads MyOne streptavidin T1 for 30 min at room temperature with low vortexing. Beads were captured with a magnet and washed twice before cDNA synthesis with the SuperScript VILO kit. (Invitrogen) and quantitative PCR (qPCR) for gRNA (nsP2) and gRNA plus sgRNA (E2).

### RNase A protection assay.

Supernatant fluid samples from dAP-7 cells infected with SINV and treated or untreated with anti-E2 antibody were centrifuged at 1,200 × *g* for 5 min to pellet cellular debris. Cell-free supernatant fluids were collected and treated with 10 μg/mL RNase A (Thermo Fisher Scientific) for 1 h at room temperature. Following treatment, RNA was isolated from the samples with TRIzol reagent and reverse transcribed to cDNA using Maxima H minus first strand cDNA synthesis kit with oligo(dT) primers and assessed by PCR as described above.

### Defective genome cloning, *in vitro* transcription, poly(A) addition, and lentivirus plasmid expression.

The nsP1 defective genome was PCR-amplified from the cDNA of SINV-infected dAP-7 cells 24 h after infection using Easy-A PCR cloning polymerase (Agilent Technologies) and the primers SINV 1F (5′-ATTGACGGCGTAGTACACACTA-3′) and oligo(dT)_18_. The PCR products were separated by gel electrophoresis, and the band of interest was excised and purified using the QIAquick gel extraction kit (Qiagen). Following sequencing confirmation, the defective genome sequence was cloned into the pcDNA3.1/V5-His-TOPO vector (Thermo Fisher Scientific) and the plasmid was amplified. For production of RNA for transfection, the plasmid was linearized using XhoI (New England Biolabs) and *in vitro* transcribed and capped using the mMessage mMachine SP6 transcription kit (Ambion). The transcribed RNA was precipitated with LiCl and polyadenylated using poly(A) polymerase (New England Biolabs). The RNA was repurified by phenol-chloroform extraction and ethanol precipitation prior to transfection.

For transient transfections, pGenLenti plasmids were generated for transduction to express either nsP1 or DVG (Genscript). dAP7 cells were established as above and incubated with lipofectamine and 1 μg of plasmid for 24 h before confirmation of transcription and translation by qPCR and immunoblotting. Transfected cells were then infected followed by antibody treatment or not as described. Cell lysates were collected in TRIzol for quantification of E2 and nsP2 RNA by qPCR and in radioimmunoprecipitation (RIPA) buffer for protein by immunoblotting.

### RNA transfection and immunoblot analysis.

A total of 10^6^ undifferentiated AP-7 cells were transfected with 2.5 μg RNA and 10 μL of Lipofectamine 2000 reagent (Thermo Fisher Scientific). Twenty-four hours after transfection, protein lysates were collected for immunoblot analysis with radioimmunoprecipitation (RIPA) buffer (50 mM Tris-Cl [pH 8.0], 150 mM NaCl, 1% NP-40, 0.1% SDS, 0.5% Na-deoxycholate, 1 mM EDTA) containing protease and phosphatase inhibitor cocktails (Roche) as previously described (18). Protein concentration was determined using the DC assay kit (Bio-Rad). Ten micrograms of total protein were separated by SDS-PAGE, transferred to a nitrocellulose membrane (Bio-Rad), and blocked in Tris-buffered saline containing 0.1% Tween 20 (TBST) and 5% milk. Immunoblot detection was performed using primary rabbit polyclonal antibodies to nsP1, nsP2, and nsP3 (1:1,000) (63) and secondary horseradish peroxidase-conjugated goat anti-rabbit antibody (1:1,000) (Cell Signaling Technology). Membranes were developed using the Amersham ECL Prime Western blotting detection reagent (GE Healthcare).

### XRN-1 degradation assay.

Extracellular virus particles were isolated from cell culture supernatant fluids by one-step sucrose cushion ultracentrifugation, as previously described (26), or using the Viraffinity virus and viral component isolation kit (BSG). For sucrose sedimentation, 8 mL of 20% sucrose in PBS was overlaid with 22 mL of the virus-containing medium and centrifuged at 107,000 × *g* for 2 h at 4°C. For Viraffinity, virus-containing medium was added at a 4:1 ratio to the water-insoluble elastomeric polyelectrolyte and then microcentrifuged. Virus pellets were directly lysed with TRIzol reagent according to the manufacturer’s instructions for RNA analysis. Following purification, 200 μg of RNA was treated with 1 U of 5′- to 3′-exoribonuclease XRN-1 (New England BioLabs) for 1 h at 37°C to degrade noncapped RNAs. The treated RNAs were repurified by TRIzol extraction and reverse transcribed to cDNA using an Applied Biosystems high-capacity cDNA reverse transcription kit with random primers. The cDNA was assessed for SINV nsP2 and E2 gene abundance by qRT-PCR as described above.

### Statistical analysis.

Data are expressed as means ± standard deviation (SD). Statistical analyses were performed using Prism Software v8.3 (GraphPad), with a *P* value of <0.05 being considered significant. Differences between two groups were analyzed by unpaired, two-tailed Student's *t* test. Multiple comparisons between groups were made using two-way analysis of variance (ANOVA) with Sidak’s posttest.

### Data availability.

Data related to this article were deposited in SRA under BioProject no. PRJNA720710. All code can be found at https://github.com/mbartl13/mbartl13 (mbartl13/GriffinLab-Sindbis).
